# Femtosecond Laser Micropore-Enhanced Miniaturised PCB-Based Microbial Fuel Cell Biosensor for Toxicity Detection

**DOI:** 10.3390/bios16030179

**Published:** 2026-03-22

**Authors:** Tong Qi, Zhongxian Li, Hebin Sun, Wenbin Zhang, Ningran Wang, Lijuan Liang, Jianlong Zhao

**Affiliations:** 1 State Key Laboratory of Transducer Technology, Shanghai Institute of Microsystem and Information Technology, Chinese Academy of Sciences, Shanghai 200050, China; qitong@mail.sim.ac.cn (T.Q.);; 2 Center of Materials Science and Optoelectronics Engineering, University of Chinese Academy of Sciences, Beijing 100049, China; 3 School of Information Science and Technology, Shanghaitech University, Shanghai 201210, China

**Keywords:** microbial fuel cell, toxicity biosensor, PCB-based biosensor, femtosecond laser

## Abstract

This study presents a low-cost, small-scale single-chamber microbial fuel cell (MFC) toxicity biosensor fabricated on a printed circuit board (PCB) and a 3D-printed chamber with a volume of 120 μL. The anode consists of a screen-printed carbon electrode on the PCB, while the air cathode is a carbon paper electrode. To address poor adhesion of microorganisms to the smooth anode surface, femtosecond laser processing was used to fabricate a micropore array with 40 μm pores on the electrode. This method can create micropores on the anode surface without damaging the screen-printed electrodes, the PCB substrate, or the pads. These micropores increase the anode’s surface area and hydrophilicity, allowing more microbial coatings to firmly adhere to its surface. In this study, the MFC utilised *Rhizobium rosettiformans* W3, extracted from activated sludge at a wastewater treatment plant, as the anode microorganism. Its aerobic nature simplifies the design of MFCs, enabling a single-chamber structure and miniaturisation. Using formaldehyde solution as a toxicity sample to test the biosensor’s performance, a 0.1% concentration significantly reduced the sensor’s output power.

## 1. Introduction

Microbial fuel cells (MFCs) are bioelectrochemical devices that use microorganisms as catalysts. Through microorganisms’ ability to oxidise organic and inorganic matter and transfer the generated electrons to the anode, chemical or biological energy is converted into electrical energy, thereby generating an electric current [1]. While MFCs can be used to generate electricity, they also have great potential applications in analysis and sensing.

MFCs are generally classified into single-chamber and two-chamber structures [2,3,4,5]. Single-chamber MFCs employ a membrane-free structure, with the anode and cathode located within the same chamber. Their advantages include simple structure, low cost, and ease of use, making them suitable for small-scale applications [5]. A two-chamber MFC consists of an anode chamber and a cathode chamber. In the anode chamber, microorganisms convert substrates into electrical energy, while in the cathode chamber, electron acceptors are reduced. The anode and cathode chambers are separated by a cation exchange membrane [5]. Two-chamber MFCs are more efficient because they confine microorganisms to the anode chamber, preventing cathode air from directly affecting bacterial metabolism (for anaerobic bacteria).

MFC technology has great application prospects in biosensing because the electrical energy generated by microorganisms oxidising the substrate at the MFC anode is proportional to the substrate concentration [6]. For example, MFC technology can provide a quick, easy way to replace or supplement the cumbersome, time-consuming traditional methods for measuring biochemical oxygen demand (BOD) [7,8,9]. MFCs can also be used for toxicity detection or monitoring because the inhibition of the metabolic activity of electrogenic microorganisms by toxic substrates can alter the MFC’s output current or power, enabling quantitative or semi-quantitative measurement of toxicity [10]. Toxicity testing is a method used to describe the potential effects of a single compound or mixture on an organism [11]. Given the wide variety and quantity of potentially toxic substances, it is difficult to detect all of them using direct measurement methods. Some methods primarily relied on biological approaches for non-specific toxicity detection, using organisms such as small fish, protozoa, and bacteria, combined with traditional behavioural recognition and imaging techniques to assess sample toxicity [12,13]. Subsequently, the convergence of biotechnology and electronics has led to novel biosensor technologies suitable for toxicity detection or monitoring [14]. One prominent example is the bioluminescent inhibition technology of *Vibrio fischeri* [15,16], which has been widely used globally and is considered effective. MFCs are well-suited for monitoring toxic compounds in water [2]. When toxic substances enter an MFC, they typically inhibit bacterial metabolism, leading to a rapid decrease in the MFC’s output power, which is positively correlated with the concentration of the toxic substance [17,18]. This rapid, real-time response characteristic of MFC biosensors enables them to quickly issue early warnings of changes in water quality, making them particularly suitable for monitoring unknown and complex sources of toxic substances in water. MFCs can be used to detect and monitor a variety of toxic substances, including heavy metals (such as copper, mercury, and chromium) [19,20,21,22] and organic pollutants (such as formaldehyde, pesticides, and phenolic compounds) [17,23].

Despite its promising prospects, practical applications still face numerous challenges, including long startup and stabilisation times, relatively low and unstable baseline signals, sensitivity to environmental fluctuations (such as temperature), poor repeatability, susceptibility to interference, and unsuitability for field deployment [3,6]. In this work, a miniaturised and simplified MFC biosensor based on a printed circuit board (PCB) and 3D-printed structure was developed. The design employed a single-chamber structure with a PCB substrate, ensuring structural rigidity and ease of fabrication. The anode was made from screen-printed carbon paste, a low-cost and biocompatible electrode material. The MFC was inoculated with aerobic electrogenic bacteria, a characteristic that reduces its use and deployment difficulty, eliminating the need for an anaerobic environment. To further improve the biosensor’s sensitivity, response speed, and stability, micropores were fabricated on the anode surface using a femtosecond laser. This laser ablation process increases the effective surface area and porosity of the anode, enhances bacterial adhesion and biofilm formation, and may promote better substrate and mass transfer. This toxic biosensor is simple in structure, inexpensive, and easy to use.

## 2. Materials and Methods

### 2.1. Microorganisms Preparation

Electrogenic microorganisms were isolated from activated sludge at a water treatment plant. A solution containing activated sludge was injected into a conventional MFC, an excitation voltage was applied, and LB medium was added as needed. The microorganisms were further isolated from the anode surface using the streak plate method and incubated at 28–30 °C for 2–4 days. Under the same culture conditions, the purified microorganisms were cultured in suspension to obtain an electrogenic microbial culture.

### 2.2. PCB-Based MFC Design

**Figure 1:**
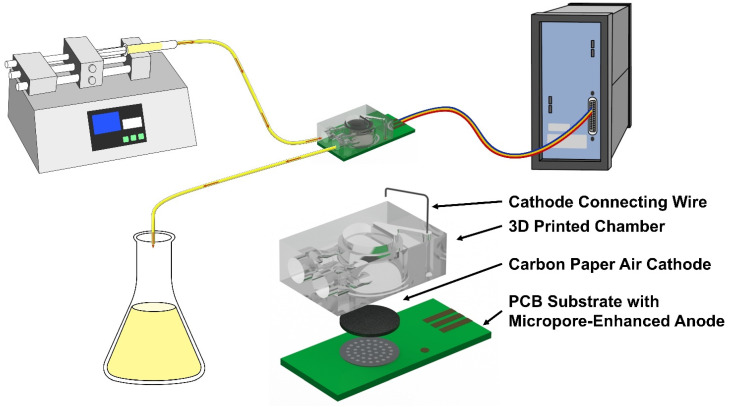
Schematic diagram of the working process and structure of the MFC-based toxicity biosensor.

**Figure 2:**
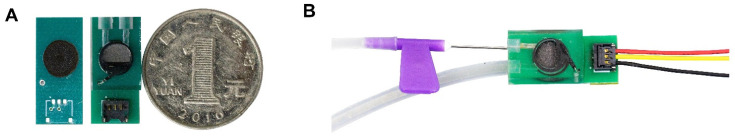
(**A**) The PCB substrate and anode (left), the assembled toxicity biosensor (middle), and its size compared with a one-yuan coin (right). (**B**) The toxicity biosensor testing and operating status.

A schematic diagram of the operation and structure of the MFC-based toxicity biosensor is shown (Figure 1). An injection pump slowly injects culture medium into the MFC, and waste liquid is then discharged. The MFC’s output voltage, current, and power are monitored. The MFC consists of a PCB serving as the anode electrode substrate and a 3D-printed solution chamber. First, a PCB with anode connection points is fabricated, and then conductive ink (Model JC101, Shanghai Julong Electronic Technology Co., Ltd., Shanghai, China) is screen-printed as the anode. Micropores with a diameter, spacing, and depth of 40 μm were etched on the anode surface using a femtosecond laser. The femtosecond laser processing equipment was manufactured by Changzhou Guangyi Laser Technology Co., Ltd. (Changzhou, China), whose model was GYET-02. The laser power, power coefficient, pulse width, beam radius, wavelength, scanning speed, and number of engravings were 5 W, 310 kJ/s, 600 fs, 10 µm, 515 nm, 800 mm/s, and twice, respectively. Epoxy resin (Model DP100, Mingnisuda Mining Manufacture Material Technology (shanghai) Co., Ltd., Shanghai, China) is used to bond the PCB to the 3D-printed structure. Torayca carbon paper (Model TGP-H-060, Toray Industries, Inc., Japan), used as the cathode, is equipped with lead wires via carbon paste adhesive and is pre-fixed to the top of the 3D printing chamber. Both the anode and cathode have a diameter of 6.6 mm. The MFC-based biosensor is 22 mm long, 10 mm wide, and 6 mm high, with a chamber volume of approximately 120 μL (Figure 2).

### 2.3. MFC Assembly and Startup

The electrogenic microbial culture was injected into the PCB-based MFC, and a 200 mV pulse was applied for 4–8 h to induce microbial biofilm growth and promote bacterial settlement under this MFC startup stage [24,25,26], while the ambient temperature was controlled at 28–32 °C. During this period, the MFC pipeline was temporarily shut down, and the air cathode was covered with a thin film to prevent evaporation of the culture medium during microbial colonisation. LB medium was then injected into the MFC at a rate of 50 μL/h using a syringe pump (Model PHD2000, Harvard Apparatus, Holliston, MA, USA), and the open-circuit potential of the MFC was continuously monitored using an electrochemical workstation (Model Reference 600, Gamry Instruments, Inc., Warminster, PA, USA) until the voltage stabilised. This process typically takes 2–4 days.

## 3. Results and Discussion

### 3.1. Structure and Anode Micropores

This study uses a PCB as the anode substrate and 3D-printed material as the reaction chamber of the MFC, combining the advantages of miniaturisation and low cost, making it suitable for toxicity biosensor applications. PCB manufacturing technology is highly mature, but PCB pads produced by conventional methods exhibit poor corrosion resistance, making them unsuitable for electrochemical systems. Therefore, gold plating on the pads has been considered to improve their corrosion resistance and reliability, while also avoiding measurement errors caused by electrochemical corrosion. However, experiments have shown that the selected conductive paste exhibits excellent water resistance. We disassembled several MFCs that had been in use for more than 2 weeks, scraped off the conductive paste to inspect the pads, and found no signs of corrosion. In the envisioned applications, the sensor will operate continuously for no more than 2 weeks, and, due to its low manufacturing cost, a replacement can be readily provided.

As a biosensor, sterile LB medium was continuously injected using a syringe pump after the MFC achieved a stable output power. Different injection speeds were tested. Injection speeds that were too fast caused the output power to drop too quickly, as the anode microorganisms might be washed away, especially immediately after bacterial inoculation, leading to sensor failure. Slow injection speeds affected the sensing response speed of the MFC. An injection rate of around 50–300 μL/h was more suitable. Therefore, we adopted a relatively low injection rate of 50 μL/L during the cultivation phase. In fact, once the MFC output stabilises, it is best to keep the MFC run at a low LB medium injection rate for 1–2 days before increasing the injection rate. But a higher sample injection rate obviously increases the liquid turnover rate within the sensor chamber, allowing the electrodes to contact the latest sample more promptly, thereby improving the overall response speed of the sensor. A conservative maximum injection rate of 300 μL/L for this sensor was confirmed through repeated experiments.

Different types of carbon paste were tested as screen printing anodes, and the one with the highest output power was ultimately selected. This carbon paste was also the most hydrophilic of the lot. Increased hydrophilicity of the anode facilitates the formation of biofilms on its surface by bacteria and enhances the transfer rate of extracellular electrons from bacteria to the electrode [27,28].

**Figure 3:**
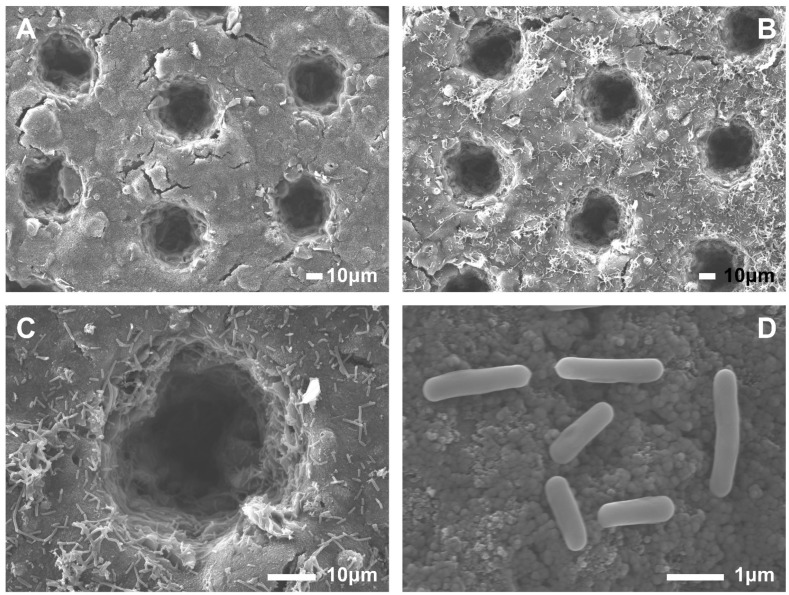
SEM images of the anode (**A**), which was made of screen-printed carbon paste, and its surface was processed using femtosecond laser technology to create micropores. The micropore diameter and depth were both 40 µm. Compared with freshly prepared anodes, the used anode surface showed microbial adhesion (**B**). Further magnification showed that the microbial density within the micropores was significantly higher than on the surface (**C**) and that the microorganisms were rod-shaped (**D**).

In this study, femtosecond laser technology was used to create micropores on the anode, increasing its surface area and roughness, thereby enhancing microbial adhesion and ultimately improving the output power and stability of the MFC. Femtosecond laser processing technology boasts extremely high peak power density, enabling precise microscale processing of materials with minimal thermal effect [29,30]. This process allows fine machining of carbon material surfaces while protecting the substrate from ablation. By controlling the depth and range of laser processing, damage to the area where the solder pads on the PCB substrate are connected to the carbon paste can be avoided, thus preventing electrode corrosion. SEM images (Figure 3) indicate that the amount of microorganisms attached to the etched micropores is more than that on the outside surface. Etched micropores provide attachment sites for microbial growth, significantly increasing the number of anodic microorganisms and helping prevent them from being washed away. Furthermore, the morphology of anodic microorganisms can also be clearly identified.

**Figure 4:**
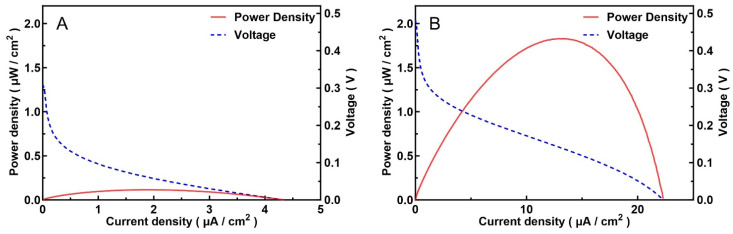
(**A**) MFC output power density using bare screen-printed carbon paste as the anode. (**B**) After fabricating micropores on the anode surface using a femtosecond laser, the output power density of the MFC.

The figure (Figure 4) shows the output power curves of the carbon paste screen-printed anode on the PCB before and after femtosecond laser etching of micropores. A significant increase in power was observed, from a maximum of 0.1 μW/cm^2^ to 1.8 μW/cm^2^ (calculated by geometric surface area), with the corresponding maximum power point voltage increasing from 60 mV to 140 mV. A higher maximum power point voltage enhances the output stability of the MFC-based biosensor.

### 3.2. Anode Microorganisms and Surface Microstructures

Since the electrogenic microorganism injected into the MFC was extracted from activated sludge, sequencing was used to identify it. 16S rRNA gene sequencing results indicate that this microorganism is *Rhizobium rosettiformans* W3 [31].

Rhizobium exhibits varying hydrophilicity and hydrophobicity under different environments [32]. SEM images reveal the morphology of this rhizobium, with a general size of 1–2 μm (Figure 3D), allowing it to adhere well to the anode micropores. This indicates good compatibility between the rhizobium and the anode material. While most rhizobia are aerobic [33], reports on *Rhizobium rosettiformans* W3 as an electrogenic microorganism are limited. However, research suggests it is indeed aerobic, which offers advantages over commonly used anaerobic electrogenic bacteria in MFC-based toxicity biosensor applications. Single-chamber MFCs can utilise these microorganisms without requiring an anaerobic environment, and the startup and use of toxic biosensors are undoubtedly relatively simple. Based on this, the study designed a single-chamber MFC, a simplified membrane-free bioreactor in which the anode and cathode are located in the same compartment. The output performance of MFC in this study was relatively low, which may be related to the electrogenic properties of *Rhizobium rosettiformans* W3 itself, but this deficiency may not be significant when used as a toxicity biosensor.

### 3.3. Toxic Responses

**Figure 5:**
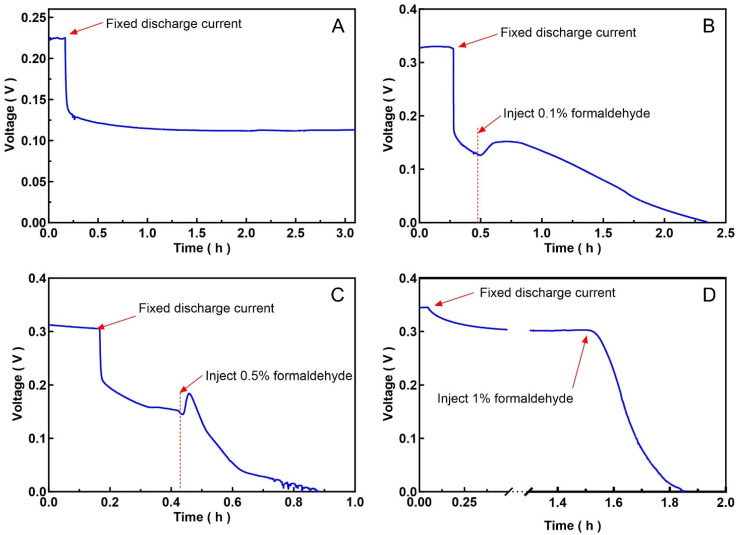
Stability testing of MFC under 100 nA load current (**A**). Formaldehyde at different concentrations was added to the biosensor to test its toxicity response (**B**–**D**).

Formaldehyde was used as a test sample to verify the performance of the MFC-based toxicity biosensor (Figure 5). During toxicity testing, a fixed discharge current of 100 nA was applied to improve stability and sensitivity. Selecting the load current requires careful evaluation, as excessive current can negatively affect the MFC output.

Formaldehyde solutions at concentrations of 0.1%, 0.5%, and 1% were tested. Experimental data showed that injecting a 0.1% formaldehyde solution at a discharge current of 100 nA induced a response in the MFC, but the process was slow, requiring 2 h for the MFC to shut down, while injecting a 1% formaldehyde solution took only about 35 min. Furthermore, the experiment found that injecting a low-concentration formaldehyde solution into the MFC resulted in a brief increase in output power, followed by a gradual decline. It is speculated that formaldehyde, as a carbon source, temporarily increased the metabolic rate of the electrogenic microorganisms, or that these microorganisms were stimulated by the toxic substance. No such anomaly was observed when a high-concentration formaldehyde solution was injected, possibly because it killed electrogenic microorganisms rapidly. But in practical applications, it is more likely to monitor the rate of decrease in sensor power output or the cumulative amount of power decrease, while ignoring other fluctuations. Furthermore, once a toxic substance enters the sensor, its output power will eventually drop to zero after a certain period. Therefore, short-term (within a few minutes) power fluctuations will not affect the sensor’s final determination of toxic substances.

Current research has limitations, making it difficult to rigorously assess the relationship between toxicity and response time. Furthermore, we speculate that when the anode microorganisms in the biosensor are killed by formaldehyde, the dead microorganisms will still adhere to the biosensor surface, occupying space. This means that even if the MFC can be restarted after re-injecting non-toxic culture medium, it cannot be restored to its original state, or even become completely unusable. While using a PCB as the electrode carrier reduces the overall cost of the sensor and simplifies mass production, the sensor can be used as a disposable device.

## 4. Conclusions

This study designed a toxicity biosensor based on an MFC, assembled using a PCB and a 3D-printed structure. A screen-printed carbon electrode anode is mounted on the PCB, while a carbon paper cathode is installed within the 3D-printed chamber, directly exposed to air, serving as an air cathode. This single-chamber MFC offers advantages such as extremely low cost and a simple manufacturing process. However, the adhesion of microorganisms to the screen-printed carbon electrode surface is insufficient, so as the culture medium is continuously injected, the anode microorganisms may be washed away, thereby affecting the output power of the MFC and causing system instability. Utilising the high precision and cold-processing capabilities of femtosecond laser technology, micron-sized micropores were fabricated on the screen-printed carbon electrode surface without damaging the PCB substrate, thereby preventing damage to the anode waterproofing and corrosion of the PCB anode pads. These micropores increase the anode’s surface area, allowing microorganisms to adhere more firmly and increasing the total number of microorganisms on the anode. The presence of micropores also improves the anode’s hydrophilicity. Tests show that the anode made of hydrophilic carbon paste outperforms the anode made of non-hydrophilic carbon paste, especially in terms of MFC start-up success rate and power output stability.

The microorganisms used in the MFC are derived from activated sludge extracted from wastewater treatment plants and identified as *Rhizobium rosettiformans* W3 by 16S rRNA gene sequencing. Due to their aerobic nature, MFC-based biosensors do not require cation-exchange membranes, allowing them to be designed very small. Final formaldehyde testing also validated that the biosensor can be used for toxicity detection. The lowest formaldehyde concentration tested so far is 0.1%.

Future work will focus on optimising response time, conducting more tests on the toxicity response of biosensors beyond formaldehyde, and enriching the functionality of it, such as adding active temperature control instead of relying on external constant temperature conditions, and adding monitoring of the internal working environment of the MFC, such as pH value, temperature, and dissolved oxygen.

## Data Availability

Dataset available on request from the authors.
